# On quantitative structure-property relationship (QSPR) analysis of physicochemical properties and anti-hepatitis prescription drugs using a linear regression model

**DOI:** 10.1016/j.heliyon.2024.e25908

**Published:** 2024-02-12

**Authors:** Abid Mahboob, Muhammad Waheed Rasheed, Aya Mohammed Dhiaa, Iqra Hanif, Laiba Amin

**Affiliations:** a Department of Mathematics, Division of Science and Technology, University of Education, Lahore, Pakistan; b Department of Pharmacy, Al-Noor University College, Nineveh, Iraq; c Department of Mathematics and Statistics, University of Agriculture, Faisalabad 38000, Pakistan

**Keywords:** Chemical compounds, QSPR analysis, Anti-hepatitis drugs, Topological indices, Regression model

## Abstract

Numerous studies demonstrate a strong intrinsic relationship between the boiling and melting temperatures, among other chemical properties, of chemical compounds and pharmaceutical and their molecular structures. Using topological indices, researchers can learn more about the physical traits, chemical stability, and bioactivities of these chemical molecular structures. Topological indices on the chemical structure of chemical materials and drugs are investigated in order to make up for the absence of chemical experiments and provide a theoretical basis for the manufacture of medications and chemical materials. According to well-known degree-based topological indices, the chemical structures of drugs used to treat hepatitis (A, B, C, D, and E) are assessed in this study. The atoms are thought of as the vertices of a graph, and the borders that separate them are thought of as the edges. Using degree-based topological indices, a quantitative structure-property relationship (QSPR) investigation was conducted to predict the physical properties of 16 hepatitis medications. These topological indices link the chemical structure to specific physical characteristics, such as the surface tension of hepatitis medication molecules and molecular weight, enthalpy, boiling point, density, vapor pressure, and logP. Using their molecular structures, the study's drugs are represented as molecular graphs, and 14 topological indices are computed.

## 1 Introduction

A significant public health issue on a worldwide scale is viral hepatitis. Hepatitis can be brought on by chemicals, drugs, some medical complications, and excessive alcohol consumption. Hepatitis A, B, C, D, E, and G infections are the six different types of hepatitis viruses that are now known. The faeces serve as the main site of infection for the hepatitis A virus (HAV) and the hepatitis E virus (HEV), with the fecal-oral pathway being the most common method of spread. Plasma viruses (Hepatitis B virus, Hepatitis C virus, and Hepatitis D virus) are most commonly spread by a percutaneous (skin-to-skin) or mucosal breach. There is information available on Hepatitis A, its classification, life cycle, clinical symptoms, missing diagnosis, and vaccination [1]. Children in various countries commonly contract acute viral hepatitis A, which accounts for 50% to 60% of all paediatric cases of acute viral hepatitis. For detail information about hepatitis B, it effect, symptoms and treatment suggestion see [2].

Usually, the hepatitis E virus is transmitted through groundwater that has been tainted by feces. For further information on the causes, history, and development of hepatitis E, read [3]. These viruses cause acute hepatitis, which is curable for 6 months in 80% of HBV patients and 20% of HCV patients. HCV is most commonly transmitted in wealthy countries through IV drug use, dialysis, hemodialysis, needle-stick wounds, piercing, sexual contact, and foetal illnesses. Infections like Hepatitis B, C, and HIV are all commonly spread through therapy treatments given with contaminated needles or syringes and insufficient sanitation of penetrating medical equipment in developing nations. Evan et al. [4] recently proposed an article on the treatment of hepatitis for those who inject drugs. Internationally, it is claimed that hazardous injection techniques and misuse lead to 8-16 million people being infected with hepatitis B, 2-5 million cases of hepatitis C, and 80 thousand to 1.5 million cases of HIV.

In this era of rapid scientific improvement, chemical and biological activities have led to quick progress, resulting in the emergence of a number of novel nanomaterials, nanocrystals, and medicines each year. QSAR modeling is a major technique for pharmaceutical research that connects molecular structure to biological activity. This efficient method enables researchers to predict a molecule's biological activity based on its chemical structure, allowing them to identify pharmacological significance more quickly and efficiently. QSAR modeling has changed the pharmaceutical industry, giving a reliable and cost-effective technique to find potential medicines. Fascinatingly, chemical research has uncovered a strong correlation between the topology of molecules and their physiological behaviors, physicochemical properties, and parameters, such as melting point, boiling point, and drug toxicity. To look into the significance of topological indices in medicinal chemistry and bioinformatics and current developments in drug discovery using networks, see [5]. In theoretical chemistry, topological indices are quantitative parameters associated with chemical compositions, physical properties, and reactivities of chemical or biological behaviors. Researchers analyzed a large number of chemical compounds and their molecular structures to draw conclusions about the properties of medicines.

The topological index is a powerful tool that takes numerical data as input and provides valuable insights into the properties of chemical compounds. There are several types of topological indices, such as degree-based, distance-based, eigen value-based, eccentricity-based, and energy-based indices. Of these, degree-based topological indices are the most widely used. The hepatitis medicines are examined with the help of a linear regression model. Furthermore, topological indices have been used to calculate and improve the physical and chemical characteristics of medicines. Theoretical chemistry focuses on the concept of chemical graphs, also known as molecular graphs, which represent molecules' atoms as vertices and their connections as edges. This helps to bridge the gap between graph theory and biochemical science. The QSPR analysis of the 16 hepatitis drugs with the 14 topological indices is done to benefit chemists and pharmacists in their understanding of the effects of drugs on the body.

There are several topological indices available today, some of which are used in biochemistry. The structural characteristics of the graphs utilized for their analysis can be used to categories them. One such index is the Hosoya index, which is determined by identifying non-incident edges in a graph. Additionally, the graph's spectrum serves as the foundation for the Estrada index. The Zagreb indices aid in understanding the branching nature of carbon atoms and pi-electron energy calculations. The ABC-index can be used to identify features of molecular structures that are connected to heat, like the heat of creation. The GA-index is important for describing the enthalpy of development and the acentric component of the hydrocarbons. Harold Wiener first utilized and described it in 1947, which enabled him to compare the boiling temperatures of several alkane isomers. Zahid Raza recently calculated the predicted values of the first Zagreb connection index in a random cyclooctatetraene chain, random polyphenyls chain, and random chain network of l, octagons, hexagons, and pentagons [6].

Recently, Mehran et al. suggested that hyper Zagreb (0.786) has a predictive ability for determining the molar reactivity of anxiety drugs. He revealed that the harmonic index may accurately predict the polarization ability (0.912) of anxiety medicines [7]. Javame et al. determined the fundamental nature of degree-based topological indices and polynomials for a specific class of antiviral medicines [8]. Sheikholeslami et al. have used multiplicative degree-related indices to cancer therapy drugs [9]. Recently, Zhang et al. applied the degree-related topological indices and a linear regression model to study the properties of the malaria drugs. The QSAR study of malaria drugs shows that the first Zagreb index has highest correlation with boiling points (0.961), while the harmonic index is applicable for estimating the molar refraction with correlation values 0.963 [10].

This manuscript focuses on the drug analysis of many types of hepatitis drugs. There are 16 two-dimensional structures that are investigated using degree-based methodology. We use 14 indices and note their correlation coefficients to examine the properties of 16 anti-hepatitis medications. The analysis shows that the harmonic index can be used to determine the molar volume and molar refraction. The boiling point and flashing point can be determined using the first Zagreb index. The Randic index is critical for determining LogP readings, and the second Zagreb index has a strong relationship with the enthalpy of all 16 hepatitis drugs. As a result, the second Zagreb index can be used to calculate the enthalpy of vaporization for all hepatitis drugs. When a pharmacist produces a medicine for any drug, he must first of all be aware of the properties, such as boiling point, molar weight, flash point, etc., of the medicine's structure. This article provides pharmacists with a theoretical method for obtaining information about hepatitis drugs. This article discusses hepatitis drugs, but the same method can be used to understand the properties of other medicines such as blood cancer, heart attack, breast cancer, asthma drugs, and so on.

### 1.1 Contribution

Our study's contribution to this manuscript is summarized as follows:

– The degrees of all the two-dimensional structures are computed with the help of the edge partitioning technique. The outcomes of the edge partitioning serve as the inputs of the topological formulas. All the structures are analyzed using the 14 topological functions. For the first time, topological indices are being used to investigate hepatitis medicines.
– MATLAB is a unique software that is applied for computation verification. The experimental data for the article is collected from ChemSpider.
– The further results of statistical work, such as linear regression equations and basic parameters, are calculated with the SPSS software. Simple mathematical techniques, as well as chemical studies, have already revealed the properties of some hepatitis drugs. We first try to determine the properties with a statistical approach.
– The major part of the computations is the correlation coefficient, whose values describe the validity of the theoretical experiments. These values are useful for chemists and pharmacists in the production of hepatitis disease medicines.
– The relationship between estimated and experimental values of the properties of hepatitis medicine is described in both a numerical and graphical way to give a clear and easy understanding of the theoretically calculated values of drugs.

### 1.2 Organization of the proposed study

The remaining part of the paper is organized as follows. The Section 2 offers basic definitions and a concise overview of the literature on the terms used in this article. The Section 3 delves into the methodology and techniques employed. The Section 4 contains information on the 2D structures of hepatitis drugs, experimental values, and computations of the topological indices. The fifth and sixth Sections 5 and 6 focus on statistical computations. Section 7 compares the correlation coefficients between topological indices and the physicochemical properties of hepatitis drugs both numerically and graphically. Section 8 presents a comparative analysis of the $r^{2}$ results obtained from topological indices for hepatitis drugs and other medications utilized in the treatment of anti-tuberculosis and breast cancer. Finally, Section 9 discusses the results and provides concluding remarks. The references are mentioned in the last of article.

## 2 Some basic definitions

In this article, we use a finite connected simple graph G. The term “Adjacent” refers to a pair of vertices of G that are joined through an edge. If two vertices of a graph G are adjacent, then adjacency is indicated as *x* ∼ *y*. The connection of two vertices with a line forms an edge that is denoted by e=xy. The cardinality of vertices G that are adjacent to a particular vertex *x*, equals the “degree” of this vertex, which is represented by the symbol $d_{x}(G)$ or just by $d_{x}$. The idea of a degree in graph theory is comparable to the chemical concept of valence. A real number formed from a graph that must be structurally invariant is known as a topological index. In 1947, Harold Wiener developed the Wiener index and used it to ascertain the physical characteristics of classes of alkanet referred to as paraffin. The use of topological indices started at this time.

### 2.1 First, second, and third Zagreb index

Gutman and Trinajstic used a numerical method to investigate the effect of total pi-electron energy on molecule structure. The well-known Zagreb index, which was first published in 1972 [11] and then expanded upon in 1975 [12], is one of the earliest graph invariants. For a graph G, the first, second, and third Zagreb indices ($Z_{1}$, $Z_{2}$, and $Z_{3}$) are defined as follows:

$$Z_{1}(G)=\sum_{xy\in E(G)}(d_{x}+d_{y}),$$

$$Z_{2}(G)=\sum_{xy\in E(G)}(d_{x}\times d_{y}),$$

$$Z_{3}(G)=\sum_{xy\in E(G)}|d_{x}-d_{y}|.$$

Here, $d_{x}$ represents the degree of vertex *x*. The degree of branches in the molecular carbon-atom skeleton is reflected through these topological indices. We suggest referring to [13], [14], [15], [16], [17] for details on the historical context, numerical findings, and statistical characteristics of Zagreb indices.

### 2.2 Re-defined third Zagreb index

Ranjini and Usha [18] were the first to presented the redefined Zagreb indices of graph G as degree-based topological indices in 2016. The redefined Zagreb indices are used to understand carborundum structures [19]. The redefined third Zagreb index of a connected graph is computed as:

$$ReZG_{3}(G)=\sum_{xy\in E(G)}(d_{x}\times d_{y})(d_{x}+d_{y}).$$

### 2.3 Reduced second Zagreb index

Furtula et al. [20] introduced the reduced Zagreb indices in 2014. In their study of the difference between the two Zagreb indices, they demonstrated that this distinction is strongly related to the vertex-degree-based invariant known as the reduced second Zagreb index, which is stated as:

$$RZ_{2}(G)=\sum_{xy\in E(G)}(d_{x}-1)(d_{y}-1).$$

Lately, the comparison of $Z_{1}$ and $Z_{2}$ of graphs has received a lot of interest. The Zagreb indices and its modified forms are useful for determination of different characteristics of the drugs. Straight comparison were carried out on the Zagreb indices for trees [21], [22] and cyclic graphs. Malik et al. applied the different forms of zegreb indices for the analysis of polycyclic aromatic hydrocarbons with applications [23].

### 2.4 Hyper Zagreb index

Shirdel et al. [24] introduced the hyper Zagreb index in 2013, which is a modified version of the Zagreb index. Numerous researchers have developed and investigated various modifications, generalizations, and extensions of Zagreb indices. The HZ(G) formula is defined as follows:

$$\text{HZ}(G)=\sum_{xy\in E(G)}{\left(d_{x}+d_{y}\right)}^{2}.$$

### 2.5 Atom bond connectivity index

Estrada and Torres [25] proposed a new degree based topological index in 1998. He called his index as, “atom-bond connectivity index”, which may be readily shortened to ABC. The ABC-index is highly correlated with the thermochemical properties of alkanes, particularly with their heat of formation. The formula of the ABC index is defined as:

$$\text{ABC}(G)=\sum_{xy\in E(G)}\sqrt{\frac{d_{x}+d_{y}-2}{d_{x}\times d_{y}}}.$$

Gnanaraj et al. [26] have examined a class of pain relievers known as nonsteroidal anti-inflammatory medications using degree-based topological indices, namely the ABC-index.

### 2.6 Randic index

The chemist Milan Randic proposed the Randic index of connected graph in 1975 [27]. The Randic index is often referred to as the “connectivity index” or the “Randic connectivity index”. It is the most well-known and practical formula that exhibits excellent association with several number of molecular graph properties. It is calculated as:

$$\text{RI}(G)=\sum_{xy\in E(G)}\frac{1}{\sqrt{d_{x}\times d_{y}}}.$$

One of the most useful degree-related indices is the randic index. Bokhary [28] used degree-related indices to calculate the attributes of breast cancer medicines. He observed that the randic index had the strongest connection with BP (r=0.933), EV (r=0.977), and molar refraction (r=0.987).

### 2.7 Harmonic index

Siemion Fajtlowicz [29] developed a computer program in the 1980s for automatically generating hypotheses in graph theory. Then he looked at potential connections between a huge number of graph invariants, one of which was a vertex-degree-based variable. Afterward (in 2012), Zhang [30] discovered that unknown variable and gave it the name harmonic index. On simple connected networks and trees, he found the lowest and highest values of the harmonic index and described the accompanying extremal graphs.

$$\text{H}(G)=\sum_{xy\in E(G)}\frac{2}{\left(d_{x}+d_{y}\right)}$$

Recently Sardar et al. used the harmonic index for the computation of the properties of headaches medicines [31].

### 2.8 Forgotten index

Furtula and Gutman [32] proposed the forgotten topological index in 2015. The prediction power of the forgotten index is basically equivalent to that of the first Zagreb index, and it is proposed by the inspiring the Zagreb indices. It has been demonstrated that the F index can greatly increase the physicochemical application of the First Zagreb index. The mathematical definition of F-index is defined as:

$$\text{F}(G)=\sum_{xy\in E(G)}(d_{x}^{2}+d_{y}^{2}).$$

### 2.9 Geometric arithmetic index

Vukicevic and Furtula [33] presented the geometric arithmetic index in 2009. The GA-index is an advanced version of Randic index. It is noticed that the predictive ability of the GA index is marginally superior to the predictive power of the R-index for physicochemical variables like entropy, enthalpy of vaporization, standardized enthalpy of vaporization, enthalpy of formulation, and acentric factor. The mathematical definition of GA-index is:

$$\text{GA}(G)=\sum_{xy\in E(G)}\frac{2\sqrt{d_{x}\times d_{y}}}{d_{x}+d_{y}}.$$

### 2.10 Sum connectivity index

Zhou and Trinajstic [34] were the first to think about the sum connectivity index in 2009. The work of R-index serves as an inspiration for the SC-index. The implementation of the SC-index was examined in 2011 [35].

$$\text{SCI}(G)=\sum_{xy\in E(G)}\frac{1}{d_{x}+d_{y}}.$$

### 2.11 Inverse sum index

Vukicevic proposed the inverse sum index in 2010 [36]. In 2015 [37], extreme values of the IS-index were found for a variety of graph classifications, including linked graphs, molecular graphs, trees, and chemical trees, which is defined as:

$$\text{IS}(G)=\sum_{xy\in E(G)}\frac{d_{x}d_{y}}{d_{x}+d_{y}}.$$

### 2.12 Symmetric division index

Vukievi and Gaperov introduced the symmetric division index in 2010. In an effort to improve the numerous QSPR/QSAR investigations, explored a new class of molecular descriptors made up of 148 descriptors called the “discrete Adriatic indices,” but they discovered that only a small number of the descriptors in this class are useful. The SDD-index is a helpful discrete Adriatic index. Out of all the molecular descriptors now in use, this index has the best correlation capability for calculating the total surface area of poly-chlorobiphenyls. The SDD-index is determined as:

$$\text{SDD}(G)=\sum_{xy\in E(G)}(\frac{d_{x}}{d_{y}}+\frac{d_{y}}{d_{x}}).$$

## 3 Techniques used for computation of results

In this section, several techniques and approaches are employed. To perform the calculations, we used multi-functional process skills, edge partition methodology, analytical techniques, theoretical graph utilities, and degree counting methods. The correlation coefficients among T-indices and hepatitis drug characteristics were calculated using the SPSS statistical software. The CamSketch programme is excellent for the 2D sketching of molecular molecules. For mathematical equations and verification, MATLAB software has also been used. To draw the 2D graphs of comparisons between topological indices and properties of compounds, Microsoft Excel is used.

## 4 Two dimensional structures of hepatitis drugs

**Figure 1:**
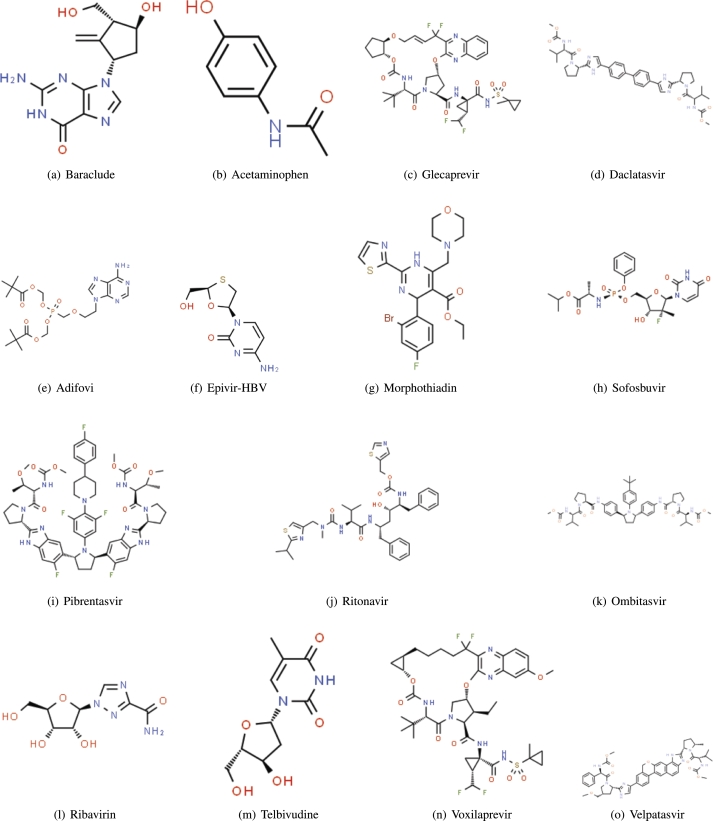
Structures of Anti-Hepatitis Drugs.

In this article, topological indices for the chemical structures of drugs prescribed to treat hepatitis disease are generated. The QSPR analysis of the indices used in the investigation is addressed, and it is determined that there is a significant relationship between the indices and the physical properties of the medicines. The 16 structures of anti-hepatitis medicines shown in Fig. 1(a-o) are analyzed using the 14 indices described in this manuscript. Although this article discusses the planar and 2D forms, drug structures (graphs) are 3D. Table 1, Table 2 provide the experimental values for the 11 physical and chemical parameters. The data in Table 1, Table 2 is not entirely complete. For the Baraclude structure, some pharmacological variables like BP, VP, EV, and FP are absent. In properties like density, molar refraction, molar volume, and surface tension, various parameters have sharp values, hence the error or extreme values. The structures are simple graphs with a maximum degree value of 4. The structure of hepatitis medications contains no vertex with a degree greater than four. The maximum size of the sample is 16 for the determination of properties, and the minimum sample size is nine, as given in Table 1, Table 2. A larger sample size is more effective in the correlation method.

**Table 1:** Physicochemical Properties of Hepatitis Drugs.

Drug name	Boiling Point	Density	Vapor Pressure	Enthalpy	Flash Point	Molar Refraction
Baraclude	-	1.8±0.1	-	-	-	67.6±0.5
Acetaminophen	387.8±25	1.3±0.1	0.0±0.9	66.2±3.0	188.4±23.2	42.4±0.3
Glecaprevir	-	1.5±0.1	-	-	-	198.2±0.4
Daclatasvir	1071.2±65	1.3±0.1	0.0±0.3	157.4±3	601.7±34.3	200.8±0.3
Adifovir Dipivoxil	641.0±65	1.4±0.1	0.0±1.9	94.6±3.0	341.5±34.3	121.0±0.5
Epivir-HBV	475.4±55	1.7±0.1	0.0±2.7	85.2±6.0	241.3±31.5	54.1±0.5
Morphothiadin	577.2±60	1.6±0.1	0.0±1.6	86.4±3.0	302.9±32.9	120.9±0.5
Sofosbuvir	-	1.4±0.1	-	-	-	123.5±0.4
Pibrentasvir	-	1.4±0.1	-	-	-	284.5±0.3
Ritonavir	947.0±65	1.2±0.1	0.0±0.3	144.4±3	526.6±34.3	198.9±0.3
Ombitasvir	1065.6±65	1.2±0.1	0.0±0.3	156.5±3	598.2±34.3	248.1±0.3
Ribavirin	639.8±65	2.1±0.1	0.0±2.0	99.3±3.0	340.7±34.3	51.1±0.5
Telbivudine	-	1.5±0.1	-	-	-	55.8±0.3
Tenofovir alafenamide	640.4±65	1.4±0.1	0.0±1.9	94.5±3.0	341.1±34.3	121.8±0.5
Voxilaprevir	-	1.4±0.1	-	-	-	207.5±0.4
Velpatasvir	-	1.3±0.1	-	-	-	-

**Table 2:** Physicochemical Properties of Hepatitis Drugs.

Drug name	IR	LogP	Molar Volume	Molar Wieght	Surface Tension
Baraclude	1.837	-0.96	153.0±7	277.279	84.2±7
Acetaminophen	1.619	0.34	120.9±3	151.163	52.8±3
Glecaprevir	1.610	1.71	571.5±5	838.865	64.6±5
Daclatasvir	1.595	5.44	591.1±3	738.875	55.4±3
Adifovir Dipivoxil	1.569	2.45	369.1±7	501.470	51.2±7
Epivir-HBV	1.755	-0.71	132.2±7	229.256	79.3±7
Morphothiadin	1.678	3.92	320.6±7	509.392	54.0±7
Sofosbuvir	1.573	1.62	374.6±5	529.453	58.7±5
Pibrentasvir	1.614	8.71	816.6±3	1113.18	57.6±3
Ritonavir	1.600	5.28	581.7±3	720.944	53.7±3
Ombitasvir	1.595	6.29	730.6±3	894.109	54.3±3
Ribavirin	1.823	-2.26	117.1±7	244.205	106.8±7
Telbivudine	1.584	-1.11	166.8±3	242.229	62.3±3
Tenofovir alafenamide	1.630	2.20	342.2±7	476.466	55.1±7
Voxilaprevir	1.596	3.83	610.3±5	868.934	61.5±5
Velpatasvir	1.643	6.78	671.6±3	883.002	60.1±3

There are 14 degree-based topological indices such as the first Zagreb index, second Zagreb index, third Zagreb index, redefined third Zagreb index, reduced second azgreb index, hyper zahreb index, atom bond connectivity index, randic index, harmonic index, forgotten index, geometric arithmetic index, sum connectivity index, inverse sum index, and symmetric division index, respectively, computed in Table 3, Table 4. All the results related to the topological indices are positive. The redefined Zagreb index values are higher than all the other indices for the same structures of the hepatitis drugs. The edge partition technique is used for the computation of the degree-based topological indices. Each index is based on vertex degrees. There are 8 different types of edge partitions present in 16 drug structures. The possible edge partitions in all the structures are given below:

$$E_{\left(1,2\right)}=\{e=xy\in E(G)|d_{x}=1,d_{y}=2\}E_{\left(1,3\right)}=\{e=xy\in E(G)|d_{x}=1,d_{y}=3\}E_{\left(1,4\right)}=\{e=xy\in E(G)|d_{x}=1,d_{y}=4\}E_{\left(2,2\right)}=\{e=xy\in E(G)|d_{x}=2,d_{y}=2\}E_{\left(2,3\right)}=\{e=xy\in E(G)|d_{x}=2,d_{y}=3\}E_{\left(2,4\right)}=\{e=xy\in E(G)|d_{x}=2,d_{y}=4\}E_{\left(3,3\right)}=\{e=xy\in E(G)|d_{x}=3,d_{y}=3\}E_{\left(3,4\right)}=\{e=xy\in E(G)|d_{x}=3,d_{y}=4\}$$

**Table 3:** Topological Indices of Hepatitis Drugs.

Drug name	Z_1_(G)	Z_2_(G)	Z_3_(G)	ReZG_3_(G)	RZ_2_(G)	HZ(G)	ABC(G)
Baraclude	110	135	18	718	47	566	15.005
Acetaminophen	50	53	12	248	14	230	8.10
Glecaprevir	322	390	70	2120	128	1692	45.68
Daclatasvir	286	341	44	1750	114	1424	42.06
Adifovir Dipivoxil	172	194	42	1020	57	806	25.94
Epivir-HBV	76	88	14	440	28	370	11.45
Morphothiadin	166	195	22	976	64	558	25.73
Sofosbuvir	187	150	45	1134	67	697	27.86
Pibrentasvir	436	533	64	2780	185	2054	62.32
Ritonavir	248	280	44	1366	85	1182	38.25
Ombitasvir	338	399	56	2048	131	1678	50.27
Ribavirin	88	105	16	550	35	444	13.7124
Telbivudine	88	104	18	540	34	442	13.60
Tenofoviralafenamide	168	435	34	970	59	615	25.40
Voxilaprevir	332	399	78	2200	139	1776	46.49
Velpatasvir	360	439	52	2284	152	1822	51.65

**Table 4:** Topological Indices of Hepatitis Drugs.

Drug name	RI(G)	H(G)	F(G)	GA(G)	SCI(G)	IS(G)	SDD(G)
Baraclude	9.52	4.55	296	21.22	9.96	25.96	51.33
Acetaminophen	5.18	2.45	124	10.47	5.18	11.45	27
Glecaprevir	26.78	12.66	912	57.55	28.15	73.80	156.16
Daclatasvir	25.98	12.53	742	57.28	23.94	68.03	134
Adifovir Dipivoxil	11.95	7.33	484	32.86	15.96	45.55	92.58
Epivir-HBV	7.202	3.466	194	15.49	7.41	17.96	36.66
Morphothiadin	15.56	7.583	418	40.23	16.23	39.41	77
Sofosbuvir	16.89	7.935	515	35.98	17.30	40.62	96.08
Pibrentasvir	37.705	18.266	1068	66.15	39.94	104.35	192.16
Ritonavir	23.97	11.53	622	51.26	24.66	58.45	122.33
Ombitasvir	31.18	14.93	880	67.75	32.20	79.68	101.44
Ribavirin	8.0409	3.816	234	17.26	8.24	76.56	43
Telbivudine	8.024	3.8	234	15.22	8.23	20.46	43.33
Tenofoviralafenamide	15.66	7.46	442	33.56	16.11	38.9	83.91
Voxilaprevir	27.23	12.72	962	62.65	28.42	75.50	140.08
Velpatasvir	31.45	15.24	944	71.18	33.23	86.25	162.66

## 5 Regression models

Data from the table above displays values that are regularly distributed. The regression analysis can be used to obtain the linear regression results for each topological indices as shown below:

(1) P(Properties)=x+y(TI),

where *P* stands for the physical or chemical characteristic of the anti-hepatitis medicine, *x* is a constant, *y* is the regression coefficient, and TI stands for the topological index. The term *x*, a constant, equals the value of P when the value of TIs = 0. All values for topological indices are positive and none have 0 values. The slope of the regression line, or *y*, indicates how much P changes for each change in the topological index TI. The experimental values of chemical and physical properties are represented by the dependent parameter *P*. The estimated values of the properties are expressed by the variable TI, an independent parameter in the equation. The effectiveness of the calculations is shown by the higher values of the correlation coefficient between the estimated values and experimental values of the hepatitis medicines. All the computations related to the linear regression given below are significant. The results of the fourteen topological indices and eleven physical parameters of hepatitis medication were determined using the SPSS software. The calculation and assessment of all the statistical information are made much easier by this software. The various linear models for the specified degree-based topological indices can be obtained using (5.1), and they are listed below. The regression equation for the 14 degree-based indices and the 11 properties of the drugs used to treat hepatitis is presented below in subsections 5.1-5.14.

### 5.1 First Zagreb index Z_1_(G)

$$\text{Boiling Point}=288.924+2.415[Z_{1}(G)]\text{Density}=1.689-0.001[Z_{1}(G)]\text{Vapour Pressure}=2.479-0.007[Z_{1}(G)]\text{Enthalpy}=51.2454+0.3249[Z_{1}(G)]\text{Flash Point}=128.555+1.461[Z_{1}(G)]\text{Molar Refraction}=4.316+0.6624[Z_{1}(G)]\text{Index of Refraction}=1.715-0.0003[Z_{1}(G)]\text{LogP}=-2.334+0.024[Z_{1}(G)]\text{Molar Volume}=-3.079+1.960[Z_{1}(G)]\text{Molar Weight}=46.295+2.474[Z_{1}(G)]\text{Surface Tension}=73.642-0.0487[Z_{1}(G)]$$

### 5.2 Second Zagreb index Z_2_(G)

$$\text{Boiling Point}=410.68+1.3154[Z_{2}(G)]\text{Density}=1.669-0.0007[Z_{2}(G)]\text{Vapour Pressure}=2.0039-0.0029[Z_{2}(G)]\text{Enthalpy}=70.935+0.1655[Z_{2}(G)]\text{Flash Point}=202.195+0.7955[Z_{2}(G)]\text{Molar Refraction}=22.807+0.4615[Z_{2}(G)]\text{Index of Refraction}=1.7042-0.0002[Z_{2}(G)]\text{LogP}=-1.7663+0.0169[Z_{2}(G)]\text{Molar Volume}=50.736+1.3816[Z_{2}(G)]\text{Molar Weight}=113.571+1.7456[Z_{2}(G)]\text{Surface Tension}=73.330-0.0381[Z_{2}(G)]$$

### 5.3 Third Zagreb index Z_3_(G)

$$\text{Boiling Point}\;=283.953+13.697[Z_{3}(G)]\text{Density}=1.691-0.0056[Z_{3}(G)]\text{Vapour Pressure}=2.435-0.0353[Z_{3}(G)]\text{Enthalpy}=53.259+1.779[Z_{3}(G)]\text{Flash Point}=125.546+8.284[Z_{3}(G)]\text{Molar Refraction}=17.539+3.177[Z_{3}(G)]\text{Index of Refraction}=1.734-0.0023[Z_{3}(G)]\text{LogP}=-1.356+0.1037[Z_{3}(G)]\text{Molar Volume}=25.467+9.957[Z_{3}(G)]\text{Molar Weight}=73.674+12.783[Z_{3}(G)]\text{Surface Tension}=74.042-0.276[Z_{3}(G)]$$

### 5.4 Re-defined third Zagreb index ReZG_3_(G)

$$\text{Boiling Point}=302.736+0.3972[ReZG_{3}(G)]\text{Density}=1.659-0.0001[ReZG_{3}(G)]\text{Vapour Pressure}=2.407-0.001[ReZG_{3}(G)]\text{Enthalpy}=55.054+0.053[ReZG_{3}(G)]\text{Flash Point}=136.912+0.2402[ReZG_{3}(G)]\text{Molar Refracttion}=14.294+0.099[ReZG_{3}(G)]\text{Index of refraction}=1.707-0.00005[ReZG_{3}(G)]\text{LogP}=-1.867+0.0034[ReZG_{3}(G)]\text{Molar Volume}=27.074+0.295[ReZG_{3}(G)]\text{Molar Wieght}=79.197+0.377[ReZG_{3}(G)]\text{Surface Tenion}=71.978-0.0067[ReZG_{3}(G)]$$

### 5.5 Reduced second Zagreb index RZ_2_(G)

$$\text{Boiling Point}=317.016+6.119[RZ_{2}(G)]\text{Density}=1.6448-0.0021[RZ_{2}(G)]\text{Vapour Pressure}=2.378-0.017[RZ_{2}(G)]\text{Enthalpy}=56.722+0.808[RZ_{2}(G)]\text{Flash Point}=145.549+3.701[RZ_{2}(G)]\text{Molar Refraction}=18.895+1.528[RZ_{2}(G)]\text{Index of Refraction}=1.699-0.0006[RZ_{2}(G)]\text{LogP}=-1.747+0.0534[RZ_{2}(G)]\text{Molar Volume}=44.340+4.4515[RZ_{2}(G)]\text{Molar Weight}=101.538+5.672[RZ_{2}(G)]\text{Surface Tension}=71.169-0.095[RZ_{2}(G)]$$

### 5.6 Hyper Zagreb index HZ(G)

Boiling Point=324.393+0.483[HZ(G)]Density=1.658−0.0001[HZ(G)]Vapour Pressure=2.396−0.0014[HZ(G)]Enthalpy=56.893+0.065[HZ(G)]Flash Point=150.00+0.292[HZ(G)]Molar Refraction=19.454+0.125[HZ(G)]Index of Refraction=1.704−0.00006[HZ(G)]LogP=−1.578+0.004[HZ(G)]Molar Volume=42.466+0.3663[HZ(G)]Molar Weight=103.97+0.462[HZ(G)]Surface Tension=70.974−0.0076[HZ(G)]

### 5.7 Atom bond connectivity index ABC(G)

Boiling Point=278.069+16.367[ABC(G)]Density=1.708−0.0076[ABC(G)]Vapor Pressure=2.520−0.0448[ABC(G)]Enthalpy=51.733+2.1539[ABC(G)]Flash Point=121.988+9.898[ABC(G)]Molar Refraction=−2.074+4.7078[ABC(G)]Index of Refraction=1.7207−0.0024[ABC(G)]LogP=−2.636+0.170[ABC(G)]Molar Volume=−23.062+13.979[ABC(G)]Molar Weight=24.130+17.542[ABC(G)]Surface Tension=74.692−0.3645[ABC(G)]

### 5.8 Randic index RI(G)

Boiling Piont=298.334+25.984[RI(G)]Density=1.7047−0.0125[RI(G)]Vapour Pressure=2.514−0.0740[RI(G)]Enthalpy=53.813+3.457[RI(G)]Flash Point=134.247+15.715[RI(G)]Molar Refraction=0.4962+7.7113[RI(G)]Index of Refraction=1.7148−0.0036[RI(G)]LogP=−2.569+0.279[RI(G)]Molar Volume=−13.672+22.786[RI(G)]Molar Weight=38.254+28.469[RI(G)]Surface Tension=74.105−0.5758[RI(G)]

### 5.9 Harmonic index H(G)

Boiling Point=284.87+54.597[H(G)]Density=1.714−0.0268[H(G)]Vapour Pressure=2.522−0.1519[H(G)]Enthalpy=52.426+7.212[H(G)]Flash Point=126.102+33.019[H(G)]Molar Refraction=−2.753+16.314[H(G)]Index of Refraction=1.7189−0.008[H(G)]LogP=−2.743+0.598[H(G)]Molar Volume=−23.159+48.135[H(G)]Molar Weight=27.580+60.011[H(G)]Surface Tension=74.922−1.2796[H(G)]

### 5.10 Forgotten index F(G)

Boiling Point=290.024+0.927[F(G)]Density=1.687−0.0003[F(G)]Vapour Pressure=2.446−0.0025[F(G)]Enthalpy=53.520+0.1215[F(G)]Flash Point=129.220+0.5603[F(G)]Molar Refraction=6.213+0.2465[F(G)]Index of Refraction=1.718−0.0001[F(G)]LogP=−2.108+0.008[F(G)]Molar Volume=0.419+0.7346[F(G)]Molar Weight=47.376+0.9328[F(G)]Surface Tension=73.404−0.018[F(G)]

### 5.11 Geometric arithmetic index GA(G)

Boiling Point=293.346+11.667[GA(G)]Density=1.727−0.0063[GA(G)]Vapour Pressure=2.496−0.0324[GA(G)]Enthalpy=53.889+1.532[GA(G)]Flash Point=131.228+7.056[GA(G)]Molar Refraction=−8.146+3.793[GA(G)]Index of Refraction=1.722−0.0019[GA(G)]LogP=−2.814+0.135[GA(G)]Molar Volume=−32.878+10.968[GA(G)]Molar Weight=15.183+13.680[GA(G)]Surface Tension=75.692−0.305[GA(G)]

### 5.12 Sum connectivity index SCI(G)

Boiling Point=287.792+25.714[SCI(G)]Density=1.705−0.012[SCI(G)]Vapour Pressure=2.505−0.0709[SCI(G)]Enthalpy=52.944+3.389[SCI(G)]Flash Piont=127.872+15.550[SCI(G)]Molar Refraction=−1.194+7.499[SCI(G)]Index of Refraction=1.718−0.0037[SCI(G)]LogP=−2.612+0.270[SCI(G)]Molar Volume=−17.492+22.052[SCI(G)]Molar Weight=31.069+27.674[SCI(G)]Surface Tension=74.487−0.572[SCI(G)]

### 5.13 Inverse sum index IS(G)

Boinling Point=309.312+8.399[IS(G)]Density=1.548−0.001[IS(G)]Vapour Pressure=2.134−0.0168[IS(G)]Enthalpy=56.055+1.100[IS(G)]Flash Point=140.916+5.079[IS(G)]Molar Refraction=16.450+2.382[IS(G)]Index of Refration=1.675−0.0005[IS(G)]LogP=−1.628+0.080[IS(G)]Molar Volume=32.366+7.129[IS(G)]Molar Weight=80.487+9.1908[IS(G)]Surface Tension=63.533−0.0057[IS(G)]

### 5.14 Symmetric division degree index SDD(G)

Boiling Point=252.662+5.810[SDD(G)]Density=1.695−0.002[SDD(G)]Vapour Pressure=2.539−0.0153[SDD(G)]Enthalpy=49.104+0.7558[SDD(G)]Flash Point=106.594+3.515[SDD(G)]Molar Refraction=0.545+1.4946[SDD(G)]Index of refraction=1.724−0.0008[SDD(G)]LogP=−2.460+0.054[SDD(G)]Molar Volume=−14.135+4.4214[SDD(G)]Molar Weight=24.547+5.659[SDD(G)]Surface Tension=74.730−0.119[SDD(G)]

## 6 Parameters of regression model

In a regression model, different parameters have different functions. The parameter *N* represents the number of items (population) in a sample. The value of *p* indicates the significance of the resulting data. If the value of p≤0.05 than outcome is significant otherwise not significant. The numbers *x* and *y* are the constant and coefficient of topological index, respectively. The number *r* is the correlation coefficient between the estimated and experimental values of the physical properties. The values of *r* may be positive (direct relation) or negative (inverse relation). R-squared offers an evaluation of the connection between the movements of a dependent variable and those of an independent variable. It does not indicate the accuracy of the selected model or the accuracy of the data and estimates. Table 5, Table 6, Table 7, Table 8, Table 9, Table 10, Table 11, Table 12, Table 13, Table 14, Table 15, Table 16, Table 17, Table 18 contains all of the results for the six parameters for the 14 degree based topological indices. The first six Table 5, Table 6, Table 7, Table 8, Table 9, Table 10 representing the computation of the different forms of the Zagreb indices. Almost all of the *p*-values are statistically significant because they are less than 0.05. Table 5 shows that the *p* values for the ST are not significant (p=0.134). All of the degree-based indices have non-significant *p* values for the ST. Table 6 shows that the sample size ranges from 9 to 16, the correlation coefficient ranges from −0.3962 to 0.9071, and all *p* values are significant with the exception of IR (p=0.121) and ST (p=0.128). In Table 7, the maximum and minimum values of *r* are ST (r=−0.3931) and MW (r=0.9132), respectively. Table 8 shows the data for the third Zagreb index as it has been redefined. In Table 9, the maximum correlation values are greater than 0.9, showing the reduced second Zagreb index's suitability for analyzing hepatitis medicine.

**Table 5:** Computation of parameters for first Zagreb index.

Properties	*N*	*x*	*y*	*r*	*r*^2^	*p*
BP	9	288.925	2.416	0.9462	**0.8953**	0.000
D	16	1.68	−0.001	−0.521	0.2714	0.038
VP	9	2.479	−0.006	−0.7178	0.5152	0.029
EV	9	53.13	0.139	0.9184	**0.8435**	0.000
FP	9	128.55	1.460	0.9462	**0.8953**	0.000
MR	15	4.316	0.6624	0.9854	**0.971**	0.0000
IR	16	1.71	−0.0003	−0.4556	0.2076	0.076
LogP	16	−2.334	0.024	0.8761	0.7676	0.000
MV	16	−3.079	1.960	0.9814	**0.9631**	0.000
MW	16	46.29	2.474	0.9945	**0.989**	0.000
ST	16	73.642	−0.0487	−0.391	0.1529	0.134

**Table 6:** Computation of parameters for second Zagreb index.

Properties	*N*	*x*	*y*	*r*	*r*^2^	*p*
BP	9	410.68	1.3154	0.7297	0.5325	0.025
D	16	1.669	−0.0007	−0.492	0.2421	0.052
VP	9	2.0039	−0.0029	−0.4563	0.2082	0.217
EV	9	70.935	0.1655	0.6771	0.4585	0.045
FP	9	202.195	0.7955	0.7296	0.5323	0.025
MR	15	22.807	0.4615	0.8951	**0.8012**	0.000
IR	16	1.7042	−0.0002	−0.4036	0.1629	0.121
LogP	16	−1.7663	0.0169	0.8126	0.6603	0.000
MV	16	50.736	1.3816	0.8938	0.7989	0.000
MW	16	113.571	1.7456	0.9071	**0.8228**	0.000
ST	16	73.330	−0.0381	−0.3962	0.157	0.128

**Table 7:** Computation of parameters for third Zagreb index.

Properties	*N*	*x*	*y*	*r*	*r*^2^	*p*
BP	9	283.95	13.697	0.8729	0.762	0.002
D	16	1.691	−0.0056	−0.5101	0.2602	0.043
VP	9	2.435	−0.0353	−0.6294	0.3961	0.069
EV	9	53.259	1.779	0.8356	0.6982	0.005
FP	9	125.546	8.284	0.8729	0.762	0.002
MR	15	17.539	3.177	0.8771	0.7693	0.000
IR	16	1.734	−0.0023	−0.5596	0.3132	0.024
LogP	16	−1.356	0.1037	0.6842	0.4681	0.003
MV	16	25.467	9.957	0.8856	0.7843	0.000
MW	16	73.674	12.783	0.9132	**0.8339**	0.000
ST	16	74.042	−0.276	−0.3931	0.1545	0.132

**Table 8:** Computation of parameters for redefined third Zagreb index.

Properties	*N*	*x*	*y*	*r*	*r*^2^	*p*
BP	9	302.736	0.3972	0.9469	**0.8966**	0.000
D	16	1.659	−0.0001	−0.4729	0.2236	0.064
VP	9	2.407	−0.001	−0.6961	0.4846	0.037
EV	9	55.054	0.053	0.9173	**0.8414**	0.000
FP	9	136.912	0.2402	0.9469	**0.8966**	0.000
MR	15	14.294	0.099	0.9649	**0.931**	0.000
IR	16	1.707	−0.00005	−0.4253	0.1809	0.100
LogP	16	−1.867	0.0034	0.8387	0.7034	0.000
MV	16	27.074	0.295	0.9613	**0.9241**	0.000
MW	16	79.197	0.377	0.9845	**0.9692**	0.000
ST	16	71.978	−0.006	−0.3467	0.1202	0.189

**Table 9:** Computation of parameters for reduced second Zagreb index.

Properties	*N*	*x*	*y*	*r*	*r*^2^	*p*
BP	9	317.016	6.119	0.9495	**0.9016**	0.000
D	16	1.6448	−0.0021	−0.4562	0.2081	0.075
VP	9	2.378	−0.017	−0.7032	0.4945	0.034
EV	9	56.722	0.808	0.9235	**0.8529**	0.000
FP	9	145.549	3.701	0.9495	**0.9016**	0.000
MR	15	18.895	1.528	0.9632	**0.9278**	0.000
IR	16	1.699	−0.0006	−0.3903	0.1523	0.135
LogP	16	−1.747	0.0534	0.8501	0.7227	0.000
MV	16	44.340	4.4515	0.9556	**0.9132**	0.000
MW	16	101.538	5.672	0.9779	**0.9563**	0.000
ST	16	71.169	−0.095	−0.3273	0.1071	0.226

**Table 10:** Computation of parameters for hyper Zagreb index.

Properties	*N*	*x*	*y*	*r*	*r*^2^	*p*
BP	9	324.393	0.483	0.9697	**0.9403**	0.000
D	16	1.658	−0.0001	−0.4866	0.2368	0.056
VP	9	2.396	−0.0014	−0.7445	0.5543	0.021
EV	9	56.893	0.065	0.9578	**0.9174**	0.000
FP	9	150.00	0.292	0.9697	**0.9403**	0.000
MR	15	19.454	0.125	0.9573	**0.9164**	0.000
IR	16	1.704	−0.00006	−0.4146	0.1719	0.110
LogP	16	−1.578	0.004	0.8143	0.6631	0.000
MV	16	42.466	0.3663	0.9562	**0.9143**	0.000
MW	16	103.97	0.462	0.9686	**0.9382**	0.000
ST	16	70.974	−0.0076	−0.3178	0.101	0.231

**Table 11:** Computation of parameters for atom bond connectivity index.

Properties	*N*	*x*	*y*	*r*	*r*^2^	*p*
BP	9	278.069	16.367	0.9438	**0.8908**	0.000
D	16	1.708	−0.0076	−0.5431	0.295	0.029
VP	9	2.520	−0.0448	−0.7237	0.5237	0.027
EV	9	51.733	2.1539	0.9156	**0.8383**	0.000
FP	9	121.988	9.898	0.9438	**0.8908**	0.000
MR	15	−2.074	4.7078	0.9926	**0.9853**	0.000
IR	16	1.7207	−0.0024	−0.4756	0.2262	0.062
LogP	16	−2.636	0.170	0.8935	0.7983	0.000
MV	16	−23.062	13.979	0.9893	**0.9787**	0.000
MW	16	24.130	17.542	0.9971	**0.9942**	0.000
ST	16	74.692	−0.3645	−0.4142	0.1716	0.110

**Table 12:** Computation of parameters for Randic index.

Properties	*N*	*x*	*y*	*r*	*r*^2^	*p*
BP	9	298.334	25.984	0.9462	**0.8953**	0.000
D	16	1.7047	−0.0125	−0.5455	0.2976	0.029
VP	9	2.514	−0.0740	−0.7557	0.5711	0.018
EV	9	53.813	3.457	0.9277	**0.8606**	0.000
FP	9	134.247	15.715	0.9462	**0.8953**	0.000
MR	15	0.4962	7.7113	0.9916	**0.9833**	0.000
IR	16	1.7148	−0.0036	−0.4462	0.1991	0.083
LogP	16	−2.569	0.279	0.8979	**0.8062**	0.000
MV	16	−13.672	22.786	0.9854	**0.971**	0.000
MW	16	38.254	28.469	0.9889	**0.9779**	0.000
ST	16	74.105	−0.5758	−0.3999	0.1599	0.1257

**Table 13:** Computation of parameters for harmonic index.

Properties	*N*	*x*	*y*	*r*	*r*^2^	*p*
BP	9	284.87	54.597	0.9443	**0.8917**	0.000
D	16	1.714	−0.0268	−0.5573	0.3106	0.025
VP	9	2.522	−0.1519	−0.7358	0.5414	0.024
EV	9	52.426	7.212	0.9193	**0.8451**	0.000
FP	9	126.102	33.019	0.9443	**0.8917**	0.000
MR	15	−2.753	16.314	0.9968	**0.9936**	0.000
IR	16	1.7189	−0.008	−0.4647	0.2159	0.073
LogP	16	−2.743	0.598	0.9132	**0.8339**	0.000
MV	16	−23.159	48.135	0.9915	**0.9831**	0.000
MW	16	27.580	60.011	0.9928	**0.9857**	0.000
ST	16	74.922	−1.2796	0.1792	−0.4233	0.102

**Table 14:** Computation of parameters for forgotten index.

Properties	*N*	*x*	*y*	*r*	*r*^2^	*p*
BP	9	290.024	0.927	0.9408	**0.8851**	0.000
D	16	1.687	−0.0003	−0.5101	0.2602	0.043
VP	9	2.446	−0.0025	−0.6952	0.4833	0.037
EV	9	53.520	0.1215	0.9092	**0.8266**	0.000
FP	9	129.220	0.5603	0.9408	**0.8851**	0.000
MR	15	6.213	0.2465	0.967	**0.9351**	0.000
IR	16	1.718	−0.0001	−0.4688	0.2198	0.067
LogP	16	−2.108	0.008	0.8323	0.6927	0.000
MV	16	0.419	0.7346	0.9677	**0.9364**	0.000
MW	16	47.376	0.9328	0.9869	**0.974**	0.000
ST	16	73.404	−0.018	−0.3799	0.1443	0.1478

**Table 15:** Computation of parameters for geometric arithmetic index.

Properties	*N*	*x*	*y*	*r*	*r*^2^	*p*
BP	9	293.346	11.667	0.9227	**0.8514**	0.000
D	16	1.727	−0.0063	−0.5643	0.3184	0.022
VP	9	2.496	−0.0324	−0.7177	0.5151	0.029
EV	9	53.889	1.532	0.8928	0.7971	0.001
FP	9	131.228	7.056	0.9227	**0.8514**	0.000
MR	15	−8.146	3.793	0.9799	**0.9602**	0.000
IR	16	1.722	−0.0019	−0.4616	0.2131	0.072
LogP	16	−2.814	0.135	0.8894	0.791	0.000
MV	16	−32.878	10.968	0.9744	**0.9495**	0.000
MW	16	15.183	13.680	0.9762	**0.953**	0.000
ST	16	75.692	−0.305	−0.4338	0.1882	0.093

**Table 16:** Computation of parameters for sum connectivity index.

Properties	*N*	*x*	*y*	*r*	*r*^2^	*p*
BP	9	287.792	25.714	0.922	**0.8501**	0.000
D	16	1.705	−0.012	−0.5415	0.2932	0.030
VP	9	2.505	−0.0709	−0.7128	0.5081	0.031
EV	9	52.944	3.389	0.8956	**0.8021**	0.001
FP	9	127.872	15.550	0.922	**0.8501**	0.000
MR	15	−1.194	7.499	0.9921	**0.9843**	0.000
IR	16	1.718	−0.0037	−0.4571	0.2089	0.075
LogP	16	−2.612	0.270	0.8981	**0.8066**	0.000
MV	16	−17.492	22.052	0.9862	**0.9726**	0.000
MW	16	31.069	27.674	0.9941	**0.9882**	0.000
ST	16	74.487	−0.572	−0.4107	0.1687	0.114

**Table 17:** Computation of parameters for inverse sum index.

Properties	*N*	*x*	*y*	*r*	*r*^2^	*p*
BP	9	309.312	8.399	0.8137	0.6621	0.007
D	16	1.548	−0.001	−0.1719	0.0295	0.526
VP	9	2.134	−0.0168	−0.4547	0.2068	0.219
EV	9	56.055	1.100	0.7863	0.6183	0.011
FP	9	140.916	5.079	0.8136	0.6619	0.007
MR	15	16.450	2.382	0.8279	0.6854	0.000
IR	16	1.675	−0.0005	−0.1802	0.0325	0.504
LogP	16	−1.628	0.080	0.6957	0.484	0.002
MV	16	32.366	7.129	0.8295	0.6881	0.000
MW	16	80.487	9.1908	0.8589	0.7377	0.000
ST	16	63.533	−0.0057	−0.0106	0.0001	0.970

**Table 18:** Computation of parameters for symmetric division index.

Properties	*N*	*x*	*y*	*r*	*r*^2^	*p*
BP	9	252.662	5.810	0.8743	0.7644	0.0021
D	16	1.695	−0.002	−0.4922	0.2423	0.0529
VP	9	2.539	−0.0153	−0.6436	0.4142	0.0618
EV	9	49.104	0.7558	0.8382	0.7026	0.0048
FP	9	106.594	3.515	0.8744	0.7646	0.0021
MR	15	0.545	1.4946	0.9283	**0.8617**	0.0000
IR	16	1.724	−0.0008	−0.4749	0.2255	0.0637
LogP	16	−2.460	0.054	0.8303	0.6894	0.0000
MV	16	−14.135	4.4214	0.9311	**0.8669**	0.0000
MW	16	24.547	5.659	0.9571	**0.916**	0.0000
ST	16	74.730	−0.119	−0.3992	0.1594	0.1258

Table 10 makes it clear that the hyper-Zegreb index has the strongest relationship with enthalpy and boiling point, with a value of 0.9697. The correlation values vary from ST (−0.4142) to MR (0.9926) and are shown in Table 11, with the exception of ST, where all *p* values are good. The correlation coefficient MR (0.9926) is extremely high and roughly equals the maximum correlation coefficient (*r* = 1). Table 12 shows clearly that the Randic index (*r* = 0.9916) is the best approach for determining MR values. For IR (0.073) and ST (0.102) in Table 13, the harmonic index *p* values are not statistically significant. The density, IR, and ST parameters shown in Table 14 have poor correlation values with the forgotten index (−0.5101, −0.4688, and −0.3799). The geometric arithmetic shows an inverse correlation with the four hepatitis pharmaceutical properties shown in Table 15. The 10 drug properties represented in Table 16 and the sum connectivity index have a very high positive correlation. In Table 17, Table 18, all indices demonstrate favorable correlation values and significant *p* values. In these tables, each of the bold numerical values of $r^{2}$ indicate that these are the best fit for a linear regression model between properties and indices.

## 7 Graphical and numerical analysis

**Figure 2:**
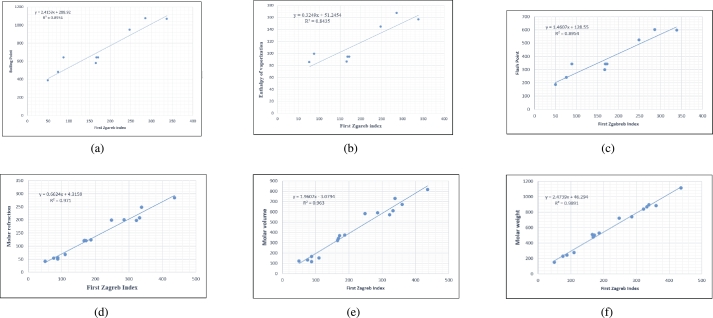
Scatterplots (with linear regression lines of best fit) demonstrated the relationship between six properties and first Zagreb index.

**Figure 3:**
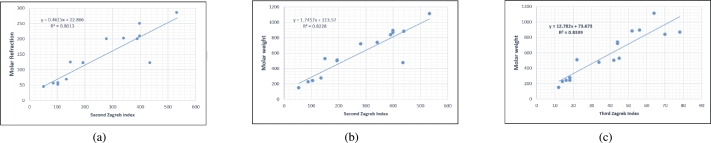
Scatterplots (with linear regression lines of best fit) demonstrated the relationship between properties and second, third Zagreb indices.

**Figure 4:**
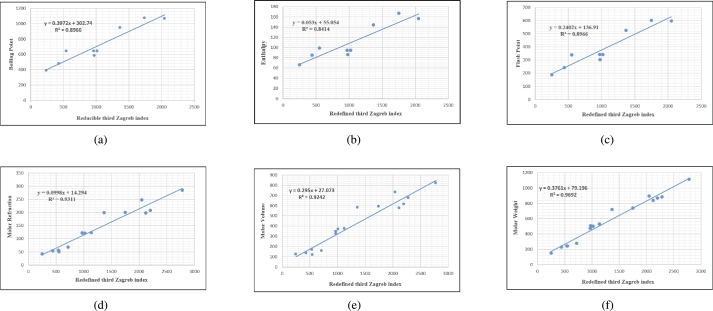
Scatterplots (with linear regression lines of best fit) demonstrated the relationship between six properties and redefined third Zagreb index.

**Figure 5:**
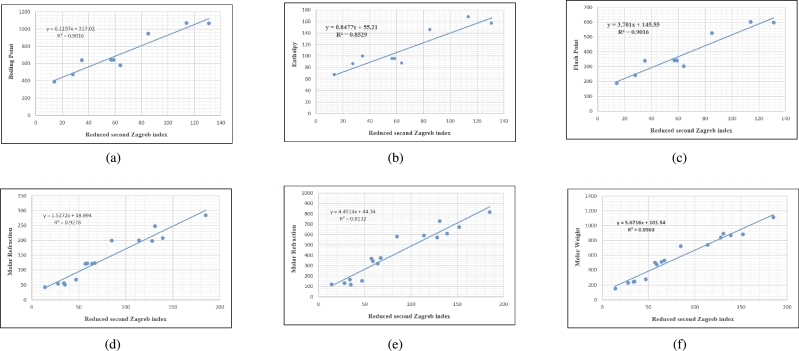
Scatterplots (with linear regression lines of best fit) demonstrated the relationship between six properties and reduced second Zagreb index.

**Figure 6:**
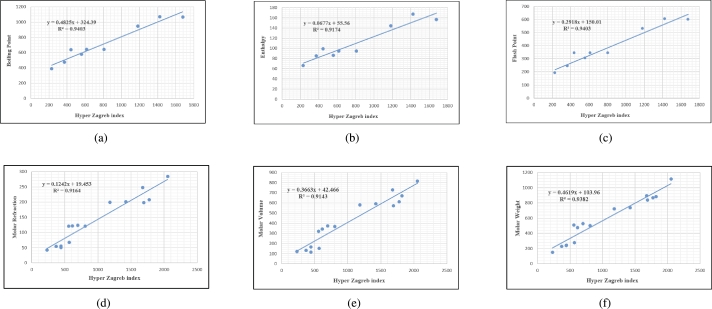
Scatterplots (with linear regression lines of best fit) demonstrated the relationship between six properties and hyper Zagreb index.

**Figure 7:**
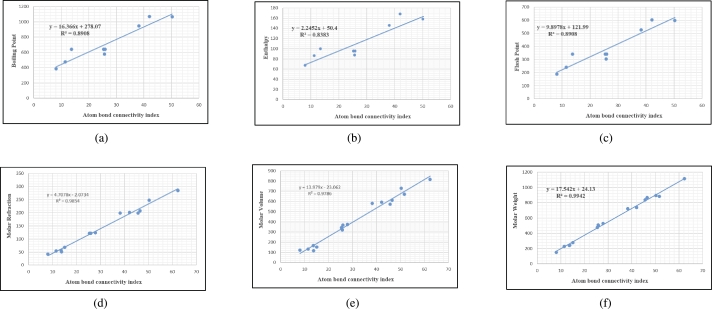
Scatterplots (with linear regression lines of best fit) demonstrated the relationship between six properties and Atom bond connectivity index.

**Figure 8:**
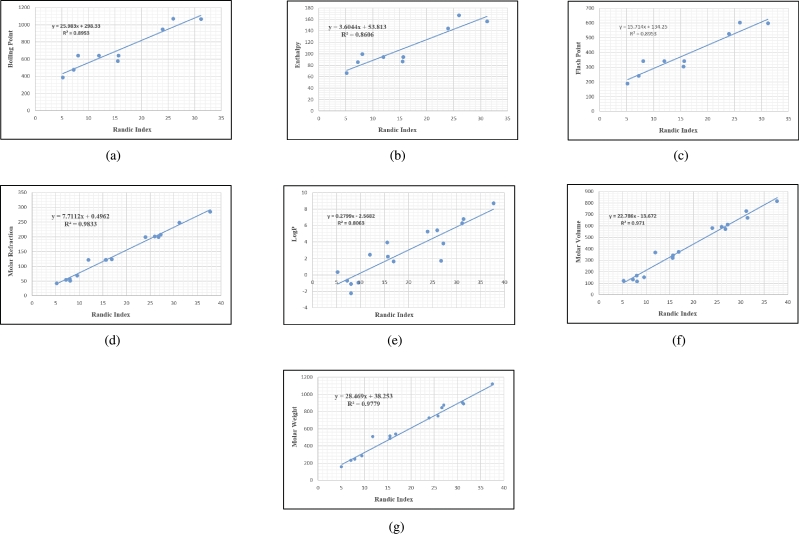
Scatterplots (with linear regression lines of best fit) demonstrated the relationship between seven properties and Randic index.

**Figure 9:**
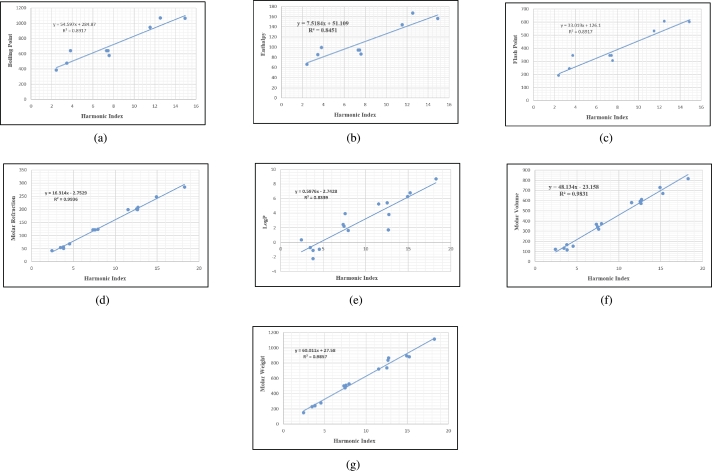
Scatterplots (with linear regression lines of best fit) demonstrated the relationship between seven properties and Harmonic index.

**Figure 10:**
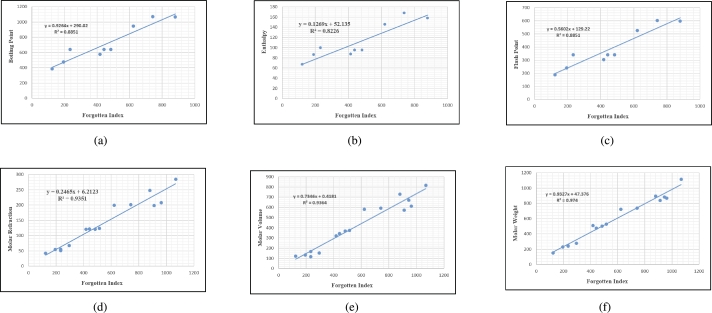
Scatterplots (with linear regression lines of best fit) demonstrated the relationship between six properties and Forgotten index.

**Figure 11:**
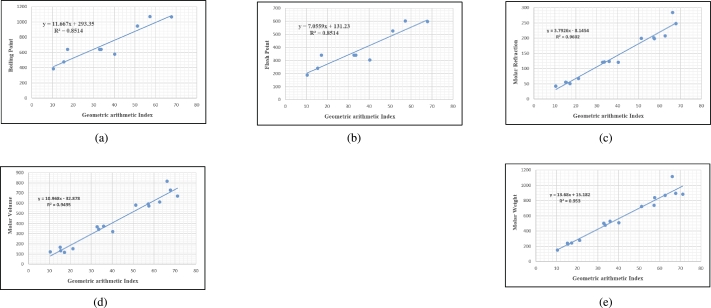
Scatterplots (with linear regression lines of best fit) demonstrated the relationship between five properties and Geometric arithmetic index.

**Figure 12:**
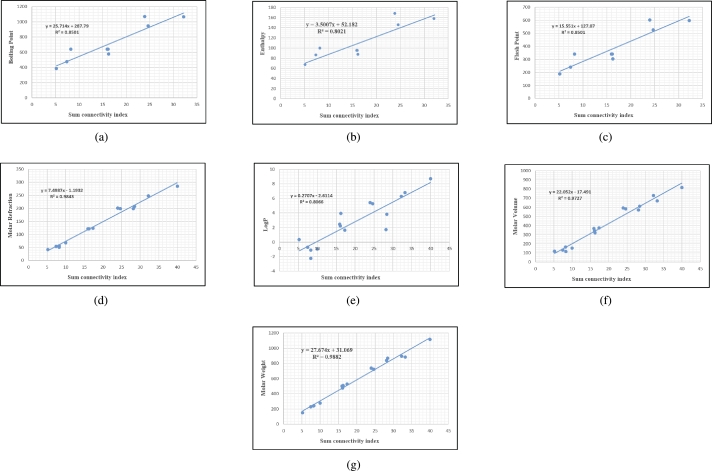
Scatterplots (with linear regression lines of best fit) demonstrated the relationship between seven properties and Sum connectivity index.

**Figure 13:**
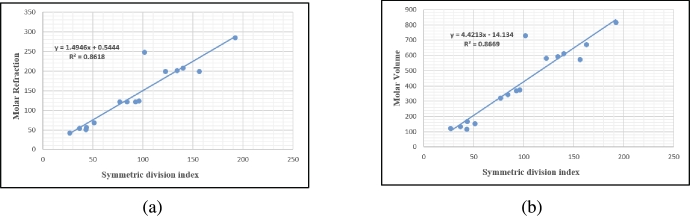
Scatterplots (with linear regression lines of best fit) illustrating the relationship between two properties and Symmetric division degree index.

**Figure 14:**
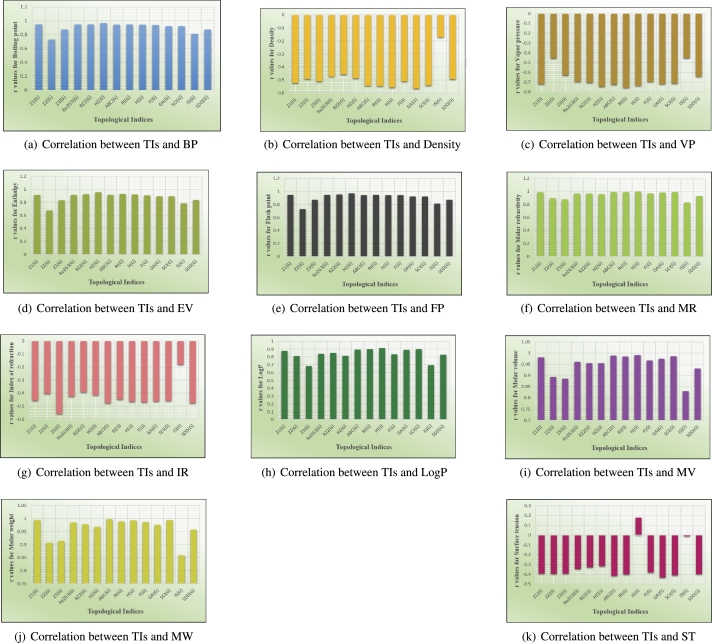
Graphical representation of correlation coefficients for all Topological indices.

Graphs are the pictorial study of data. It is simpler to interpret massive amounts of data, patterns, and linkages when the data is presented visually. Represented in Fig. 2(a-f), Fig. 3(a-c), Fig. 4(a-f), Fig. 5(a-f), Fig. 6(a-f), Fig. 7(a-f), Fig. 8(a-g), Fig. 9(a-g), Fig. 10(a-f), Fig. 11(a-e), Fig. 12(a-g) and Fig. 13(a,b) are the best fit regression lines that illustrate the relationship between properties and topological indices, with an $r^{2}$ value greater and equal than 0.8. The 2D bar graphs are used to present the comparison between the correlation coefficients of all topological indices, as shown in Figure Fig. 14(a-k). Table 19 provides a comparison of all numerical data. We utilized Microsoft Excel to draw the bar graphs for all the properties of hepatitis drugs. The data is visually represented in the bar graph through rectangular bars, with each bars height reflecting its corresponding value. Bar charts are used to track changes across time, both short and long. The line charts can also used for the comparison of the correlation coefficient. The properties with their respective high correlations are boiling point (r=0.9697), density (r=−0.5643), vapour pressure (r=−0.7557), enthalpy (r=−0.7557), flash point (r=0.9697), molar refraction (r=0.9968), IR (r=−0.5596), LogP (r=0.9132), molar volume (r=0.9132), molar weight (r=0.9779), and surface tension (r=−0.4338). The highest value of correlation is 0.9968, which shows the perfect relationship between molar refraction and harmonic index. The density, vapor pressure, IR, and surface tension graphs are drawn in the negative axis, indicating an inverse connection between topological indices and these parameters.

**Table 19:** Comparison of correlation coefficients for all topological indices.

TIs	BP	Density	VP	EV	FP	MR	IR	LogP	MV	MW	ST
Z_1_(G)	0.9462	−0.521	−0.7178	0.9184	0.9462	0.9854	−0.4556	0.8761	0.9814	0.9945	−0.391
Z_2_(G)	0.7297	−0.492	−0.4563	0.6771	0.7296	0.8951	−0.4036	0.8126	0.8938	0.9071	−0.3962
Z_3_(G)	0.8729	−0.5101	−0.6294	0.8356	0.8729	0.8771	−0.5596	0.6842	0.8856	0.9132	−0.3931
ReZG_3_(G)	0.9469	−0.4729	−0.6961	0.9173	0.9469	0.9649	−0.4253	0.8387	0.9613	0.9845	−0.3467
RZ_2_(G)	0.9495	−0.4562	−0.7032	0.9235	0.9495	0.9632	−0.3903	0.8501	0.9556	0.9779	−0.3273
HZ(G)	0.9697	−0.4866	−0.7445	0.9578	0.9697	0.9573	−0.4146	0.8143	0.9562	0.9686	−0.3178
ABC(G)	0.9438	−0.5431	−0.7237	0.9156	0.9438	0.9926	−0.4756	0.8935	0.9893	0.9971	−0.4142
RI(G)	0.9462	−0.5455	−0.7557	0.9277	0.9462	0.9916	−0.4462	0.8979	0.9854	0.9889	−0.3999
H(G)	0.9443	−0.5573	−0.7358	0.9193	0.9443	0.9968	−0.4647	0.9132	0.9915	0.9928	0.1792
F(G)	0.9408	−0.5101	−0.6952	0.9092	0.9408	0.967	−0.4688	0.8323	0.9677	0.9869	−0.3799
GA(G)	0.9227	−0.5643	−0.7177	0.8928	0.9227	0.9799	−0.4616	0.8894	0.9744	0.9762	−0.4338
SCI(G)	0.922	−0.5415	−0.7128	0.8956	0.922	0.9921	−0.4571	0.8981	0.9862	0.9941	−0.4107
IS(G)	0.8137	−0.1719	−0.4547	0.7863	0.8136	0.8279	−0.1802	0.6957	0.8295	0.8589	−0.0106
SDD(G)	0.8743	−0.4922	−0.6436	0.8382	0.8744	0.9283	−0.4749	0.8303	0.9311	0.9571	−0.3992

## 8 Analyzing the comparison of $r^{2}$ results with literature

This section presents a comparative analysis of the $r^{2}$ results obtained from topological indices for hepatitis drugs and other medications utilized in the treatment of anti-tuberculosis and breast cancer. The focus of this comparison lies in examining the outcomes generated by the linear regression parameters. The $r^{2}$ results obtained from QSPR modeling of drugs for hepatitis, anti-tuberculosis, and breast cancer are presented in Table 20. This comparison focuses on three common indices and five drug characteristics. Specifically, we examine the first Zagreb index, the Harmonic index, and the Geometric arithmetic index, as well as properties including boiling point, enthalpy of vaporization, flash point, molar refraction, and molar volume. Upon examining Table 20, it is observed that all $r^{2}$ values of hepatitis drugs for both the first Zagreb index and harmonic index lie within the range of 0.8 to 0.9. Similarly, for the geometric arithmetic index, the $r^{2}$ values fall between 0.7 and 0.9. These findings suggest a strong positive correlation between the properties and indices of the hepatitis drugs. In contrast, the correlation $r^{2}$ values of anti-tuberculosis drugs for the first Zagreb index, harmonic index, and geometric index also fall within the range of 0.7 to 0.9. This indicates a very good correlation between the properties and indices of the anti-tuberculosis drugs. At last, all $r^{2}$ values for breast cancer drugs lie within the range of 0.7 to 0.9 for both first Zagreb index and harmonic index; whereas for the geometric index is limited to a range of 0.5 to 0.8. Overall, the data from Table 20 highlights the comparison of correlation results that indicates the significant relationship between the properties and indices of hepatitis, anti-tuberculosis, and breast cancer drugs.

**Table 20:** Comparison of *r*^2^ results with literature.

T-Indices	Boiling Point	Enthalpy of Vaporization	Flash Point	Molar Refraction	Molar Volume
Results of hepatitis drugs					

Z_1_(G)	0.8953	0.8435	0.8953	0.971	0.9631
H(G)	0.8917	0.8451	0.8917	0.9936	0.9831
GA(G)	0.8514	0.7971	0.8514	0.9662	0.9495

Results of anti-tuberculosis drugs [38]					

Z_1_(G)	0.808	0.706	0.787	0.949	0.931
H(G)	0.842	0.742	0.795	0.970	0.964
GA(G)	0.799	0.748	0.779	0.826	0.841

Results of breast cancer drugs [39]					

Z_1_(G)	0.912	0.912	0.700	0.940	0.855
H(G)	0.929	0.946	0.754	0.969	0.838
GA(G)	0.688	0.788	0.576	0.803	0.775

## 9 Conclusion

The drugs prescribed to treat hepatitis are examined in the current work, and several numerical descriptors are derived for each therapy. Every innovative drug must have certain structural characteristics, which can be discovered by QSPR modeling with TIs. The purpose of this research is to use topological indices to quickly and cheaply gather information about the structure's topology. The statistical parameters for the linear QSPR models for different degree-based topological indices are easily obtained from Table 5, Table 6, Table 7, Table 8, Table 9, Table 10, Table 11, Table 12, Table 13, Table 14, Table 15, Table 16, Table 17, Table 18. Table 5, Table 6, Table 7, Table 8, Table 9, Table 10, Table 11, Table 12, Table 13, Table 14, Table 15, Table 16, Table 17, Table 18 contains the results regarding the correlation coefficients. The major outcomes about the hepatitis drugs are given below.

– The Zagreb indices are the classical and very successful indices. The first Zagreb index has the strongest and most positive values of the correlation with the BP (0.9462), EV (0.9184), FP (0.9462), MR (0.9854), MV (0.9814), and MW (0.9945). These all have strong positive correlations, which means that the first Zagreb index and these six properties have direct correlations. The $Z_{1}$-index has a negative relationship with both IR (−0.4556) and ST (−0.391). The range of the correlation values is −0.391 to 0.9945. The $Z_{2}$ index is significantly suitable for the calculation of the properties such as MV (0.9071), MV (0.8938) and logP (0.8126). The least relationship values of $Z_{2}$ is obtained with the ST (−0.3962). The IR has highest correlation with the $Z_{3}$ index.
– The $ReZG_{3}(G)$ index is suitable to calculate the BP (0.9469), EV (0.9173), FP (0.9469), MR (0.9649), MV (0.9613), and MW (0.9845). The correlation for $ReZG_{3}(G)$ ranges from −0.3467 to 0.9845. All the topological indices show very good correlation with the properties of the hepatitis drugs except ST, density, and IR.
– According to the results of the HZ index research, it has the strongest relationship with three drug properties: BP (0.9697), EV (0.9697), and FP (0.9697). The QSAR analysis of the drug studies revealed an inverse relationship between these four properties and the TIs. The correlation for this index ranges from −0.317 to 0.9697.
– The ABC index is a well-known and efficient index. The ABC-index may be used to compute molar weight since it has the greatest correlation value, MW (0.9971). The vapor pressure has a negative association with all of the TIs, and the R-index has a connection with this characteristic VP (−0.7557).
– The harmonic index is a simple index to calculate. This index has been shown to be the most meaningful index in this manuscript because of its high correlation with all degree-related topological indices. The three properties, MR (0.9968), logP (0.9132), and MV (0.9915), have the strongest relationship with the harmonic index. The maximum and minimum values of the H index correlation are MR (0.9868) and ST (0.1792). It is a unique index since it represents the correlation's extreme levels.
– Properties such as density (−0.5643) and surface tension (−0.4338) are determined with the help of the geometric arithmetic index.
– Table 19 show the correlation coefficient between topological indices and the 11 physio-chemical characteristics of the drugs. Inspection reveals that BP and HZ(G) have the strongest correlation, with r=0.9697. Also, EV and HZ(G) have the strongest correlation (r=0.9578), FP has a good correlation with HZ(G) having r=0.9697, while MR has a highest correlation with H(G) with r=0.9968 and LogP and H(G) have the strongest correlation with r=0.9132, MV has a good correlation with H(G) having r=0.9915, and MW and $RZ_{2}$(G) have the best correlation with r=0.9779.

The physical characteristics and their corresponding topological indices are well correlated in the outcomes. The analysis makes it clear that MR should have a high connection with all topological indices taken into consideration. This research suggested that theoretical assessment would enable chemists and other professionals in the pharmaceutical sector to forecast the features of hepatitis medications without performing any experiments. Given the wide range of topological indices calculated in this analysis, it is also conceivable that alternative formulations of these drugs may be utilized to treat various disorders. The correlation coefficient for several topological indices has been determined in this research, and the chemist can now use this information to create new medications by combining existing ones that have high correlations. The boiling point, density, vapor pressure, enthalpy, flash point, and molar refraction of the hepatitis medicines were investigated. The linear regression model was used in the computation. These properties are extremely complicated and difficult to calculate using laboratory experiments. The structures of hepatitis medicines are examined and analyzed using 14 degree-based indices.

### 9.1 Future work

In a similar way, the chemical and physical properties of a wide range of medicines may be calculated using various degree-based topological indices. The correlation values must be high and use particular indices for specific properties of drugs. We will attempt to analyze chemical (medicine graphs) and non-chemical graphs (computer networks) using degree, distance, and eccentricity based topological indices.

### Funding statement

This research received no funding.

## CRediT authorship contribution statement

**Abid Mahboob:** Visualization, Validation, Supervision. **Muhammad Waheed Rasheed:** Writing – review & editing, Software, Methodology, Formal analysis, Conceptualization. **Aya Mohammed Dhiaa:** Investigation, Funding acquisition, Formal analysis. **Iqra Hanif:** Writing – original draft, Software, Resources. **Laiba Amin:** Writing – original draft, Methodology, Investigation, Data curation.

## Declaration of Competing Interest

The authors declare that they have no known competing financial interests or personal relationships that could have appeared to influence the work reported in this paper.

## Data Availability

In this article, no data were utilized.
